# Universal health coverage in India and health technology assessment: current status and the way forward

**DOI:** 10.3389/fpubh.2023.1187567

**Published:** 2023-06-02

**Authors:** Chandrakant Lahariya, Krushna Chandra Sahoo, T. Sundararaman, Shankar Prinja, Kavitha Rajsekhar, Sanghamitra Pati

**Affiliations:** ^1^Integrated Department of Health Policy, Epidemiology, Preventive Medicine and Pediatrics, Foundation for People-centric Health Systems, New Delhi, India; ^2^SD Gupta School of Public Health, The IIHMR University, Jaipur, India; ^3^Health Technology Assessment in India, Regional Resource Hub, ICMR-Regional Medical Research Centre, Bhubaneswar, Odisha, India; ^4^Former Dean, School of Health Systems Studies, Tata Institute of Social Sciences, Mumbai, India; ^5^School of Public Health, Post Graduate Institute of Medical Education and Research, Chandigarh, India; ^6^Health Technology Assessment in India, Department of Health Research, Ministry of Health and Family Welfare, New Delhi, India

**Keywords:** universal health coverage, India, health technology assessment, HTA, UHC, SDG

## Abstract

In India, there is a renewed emphasis on Universal Health Coverage (UHC). Alongside this, Health Technology Assessment (HTA) is an important tool for advancing UHC. The development and application of HTA in India, including capacity building and establishing institutional mechanisms. We emphasized using the HTA approach within two components of the Ayushman Bharat programme, and the section concludes with lessons learned and the next steps. The UHC has increased the importance of selecting and implementing effective technologies and interventions within national health systems, particularly in the context of limited resources. To maximize the use of limited resources and produce reliable scientific assessments, developing and enhancing national capacity must be based on established best practices, information exchange between different sectors, and collaborative approaches. A more potent mechanism and capacity for HTA in India would accelerate the country’s progress toward UHC.

## 1. Introduction

There is renewed focus and attention on Universal Health Coverage (UHC) in India, which has been reiterated at multiple policy and programmatic initiatives such as National Health Policy 2017 (1), Ayushman Bharat Program in 2018 (2) and commitments made at global fora such as United Nation High-level meeting (UNHLM) on UHC, held in New York in Sept 2019 (3). Alongside this, the importance of Health Technology Assessment (HTA) is increasingly being recognized and is being considered an important tool to advance UHC (4).

UHC has been recognized as a global objective and is included in Agenda 2030’s Sustainable Development Goals (SDG) (5). SDG-3 is viewed as central to many other goals, and the UHC target within SDG-3 is viewed as paramount for achieving the health goal. UHC means everyone gets quality health care without financial hardship. It covers health promotion, prevention, treatment, rehabilitation, and palliative care (6). UHC ensures a progressive expansion of health services, not just a minimum package.

As financial resources are limited, when transitioning to UHC, well-considered decisions must be made regarding services to be included and covered and the appropriate delivery strategies, necessitating some form of prioritization. In moving toward UHC, questions focus on the populations covered by the package of interventions, which services can be provided and how much the proportion of service costs can be covered. In this context, Health Technology Assessment (HTA) is regarded as a crucial process for assisting with the setting of UHC priorities. The section then discusses why setting priorities is essential for countries to move toward UHC and how HTA can help. Describes the use of HTA in shaping policies in the Indian context and the Ayushman Bharat programme.

## 2. Health technology assessment: concept and evolution

HTA is the systematic assessment of the properties, effects, and/or consequences of health technologies and interventions. It examines both the intended and unintended direct and indirect outcomes of technologies and interventions. However, an unintended consequence would be unethical foetal sex determination. Similarly, antibiotics may be widely used to kill bacteria; however, unmonitored large-scale use may lead to antibiotic resistance. The HTA is utilized to inform policy and healthcare decisions, particularly regarding allocating limited funds for health interventions and technologies. HTA can be applied to interventions such as incorporating a new medicine into a reimbursement scheme, implementing large-scale public health programmes, setting priorities in health care, identifying health interventions that produce the greatest health gain and offer value for money, setting prices for medicines and other technologies based on their cost–effectiveness, and developing clinical guidelines (7, 8). HTA’s primary objective is to define policy decisions founded on scientific methods and research. HTA is equally concerned with pharmaceuticals and medical devices as well as interventions and delivery systems. For instance, it may be utilized in hospital infection control programmes, computerized drug distribution and utilization review systems, and rural telemedicine networks.

HTA itself does not make decisions; however, the systematic evaluation of evidence using HTA assists policymakers in identifying the costs and benefits of alternative actions. It differs from routine monitoring and evaluation and randomized controlled trials, which may form part of the methodology for assessing health technologies. The primary focus is on any technology or service delivery that requires evaluation for efficiency, effectiveness, safety, and consequences. Evidence-based medicine refers to the use of current best evidence from scientific and medical research (which may be derived from randomized controlled trials or other primary research designs) and the application of clinical experience and observation in making decisions regarding the care of individual patients (7, 8).

The Governments and the payers in insurance systems often use a variety of criteria for making decisions around priority setting. A recent systematic review of criteria for priority setting used in low and middle-income countries (LMICs) shows that cost-effectiveness and overall health benefits of given interventions feature as the top criteria for choosing interventions and their delivery platforms (9). To make the criteria congruous with the objectives of UHC, the impact of interventions on out-of-pocket expenditure and financial risk protection, and equity in utilization of health care services and unmet needs also become critical criteria. HTA is one of the methods to generate evidence using systematic, transparent, and robust methods.

While HTA has received increasing attention in the last decade with an overall discourse on UHC, the discussion on HTA or HITA (Health Interventions Technology Assessment) first started nearly 35 years ago in WHO and two regional offices (EURO and AMRO) (7), to strengthen evidence-based selection and rational use of health technologies. Since then, the approach has been further refined, and the application of HTA widened. A resolution on Health Technologies proposed by Mexico in May 2005 and then supported by Thailand and Netherlands was approved in the 60th World Health Assembly (WHA) in 2007 (10). It urged Member States, “to formulate as appropriate national strategies and plans for the establishment of systems for the assessment, planning, procurement, and management of health technologies in particular medical devices, in collaboration with personnel involved in health technology assessment and biomedical engineering; this concept was at that time to be applied specifically to medical devices. The WHA 60.29 also requested the Director-General to support its Member States in establishing mechanisms to assess national needs for health technologies, assure their availability and use, and implement policies on health technologies, especially for priority diseases according to different levels of care in developing countries.

Following the WHA resolution, professional societies and international and regional networks emerged to promote HTA. These include Health Technology Assessment international (HTAi), the International Network of Agencies in Health Technology Assessment (INAHTA), and the International Information Network on New and Emerging Health Technologies (EuroScan International Network) (7). In 2009 and 2010, Memoranda of Understanding were signed with these three organizations to support the implementation of the WHA60.29 Resolution on Health Technologies to disseminate the knowledge of HTA, particularly in LMICs, and evaluate innovative technologies and exchange of information (8, 9). Understanding the importance of HTA in support of UHC, a resolution (WHA67.23) was approved (7, 11–13) during the 67th World Health Assembly with major timelines and events (Box 1).

HTA is based on collecting, evaluating, and systematically reviewing all available evidence for the intervention or technology being considered. The data types include epidemiological, economic, and health impact and expert opinion. The methods include assessing the quality of available information, systematic review and meta-analysis, surveys, feasibility, affordability, and ethical considerations. HTA vary in scope, time and resources required. The final product could be a full-scale HTA report and the contextualization of reports produced by others. It studies the medical, social, ethical, and economic implications of the development, diffusion, and use of health technology. For this, multidisciplinary teams can include biomedical engineers, epidemiologists, ethicists, health economists, librarians, lawyers, nurses, patient organizations, pharmacists, and public health specialists. The five-step HTA process includes (1) Defining decision space – what is the decision problem? Topic identification and prioritization, (2) Analysis – what is the required analysis needed to help answer the decision problem? (3) Appraisal – how do we decide if the evidence is strong enough to support a decision? What are our recommendations? (4) Decision making – what is the decision to be taken? (5) Implementation – how is the decision implemented and monitored? (14).

HTA provides a decision-making framework for different decisions and can be applied in all health care systems, but needs differ (15, 16). For illustration, in low-income countries with low coverage, it can be used to decide on primary health care packages (17). In middle-income countries with limited coverage, to decide how to extend the package of health care services provided; and in systems with established UHC, to inform decisions on marginal analysis to determine which extra services to provide and at what cost (13, 18). Over the years, HTA has been used by WHO extensively, and key applications are listed in Box 2 (19–24).

**BOX 1 Global evolution of HTA at the global level (7, 11–13).**

1983–85: David Banta of the USA is credited to establish methods of HTA.

1984: The WHO European Office stated that “prior to 1990 all the Member States should have established a formal mechanism to systematically assess the appropriate use of health technologies.”

1989: AMRO/ PAHO, published information on HTA, supported workshops and provided guidance to the constitution of HTA agencies.

1991: Pan American health Organization initiated HTA concepts and publications.

1993: The Second meeting of WHO Regional Advisers on Technology Development, Assessment, and Transfer in Alexandria, Egypt.

1994: A working group, named “Promoting the Use of Health Technology Assessment to Improve Health Care in Developing Countries” met in Geneva, WHO HQ

1997: A WHO working group and two meetings on the methodology and practical applications of HTA in Mexico and Chile.

1990: Expansion of the HTA concepts and methodology in the region of the Americas; establishment of ISTAHC and

2003: Health Technology Assessment International (HTAi) was set up as a professional society

2007: A resolution on Health Technologies (WHA60.29) was approved by the 60th World Health Assembly (WHA) in 2007.

2013–2014: WHO publications and Resolutions indicate that HTA is a tool to further advance the implementation of Universal Health Coverage (UHC) in terms of deciding to get the best value for money

2015: WHO global survey of HTA use with 111-member state responses

2020–2021: An update to the survey with the component of Health Benefit Packages

**BOX 2 Examples of HTA in WHO (15–21).**

- WHO Model List of Essential Medicines: First published in 1977; Updated every 2 years and more than 160 countries have essential medicines lists. It uses the concept and approach of HTA.
- WHO-CHOICE or CHOosing Interventions that are Cost Effective global database of around 500 health technologies; Ongoing since 1998; Development of cost-effectiveness analyses of interventions covering all WHO regions
- One Health Tool for Costing and Strategic planning: Development began in 2008; Released in 2012; Has to date been used in over 40 countries
- Package of Essential Noncommunicable (PEN) disease interventions for primary health care, ‘best buys’ for NCDs
- Assessing medical devices and assistive devices for an ageing population
- The other related work of WHO in this area include the Health Technology Assessment Survey, WHO guidelines approved by the guidelines review Committee of HTA and the Development of HTA Capacity in Member states through advocacy and raising awareness on the Utility of HTA for policymakers.

## 3. Health technology assessment in India

The need and interest in HTA in India started with the release of the report of HLEG on UHC in Oct 2011 (25). Thereafter, the twelfth Five Year Plan (FYP) for India proposed to consider ‘cost-effectiveness studies to frame clinical treatment guidelines and to assess available therapies and technologies (26). During this period, in response to a question raised in Indian Parliament, a commitment was made that ‘the need to establish such a board was discussed and recommended by the 12th Plan Working Group on Health Research. The Parliamentary Standing Committee also commented that the Department of Health Research plans to focus on programmes to make healthcare affordable for marginalized groups (27). The National Health Policy, 2017 highlighted the importance of HTA by noting its importance in introducing new technologies and their uptake into public health programmes. The NHP 2017 clearly articulates the need for establishing an institutional framework along the lines of the UK’s National Institute of Clinical and Care Excellence (NICE) for carrying out HTA to guide policy (1). With this development initiative, a Medical Technology Assessment Board (MTAB) was approved (28), which later on became the Health Technology Assessment India (HTAIn) (29). Consequent to this policy recommendation, Health Technology Assessment India (HTAIn) was created with its Secretariat in the Department of Health Research. It assumed a hub-and-spoke model, in which several academic and research institutions were identified as the Regional Resource Centres (RRC) and Technical Partners (TP) to conduct assessment studies. The HTA Board is the overarching body which provides stewardship and approves the findings of an HTA study and gives recommendations to the user department.

The launch of the National Digital Health Mission (NDHM) was announced in 2020. It aimed to pursue the task of digitization of all medical records and making them centrally accessible. The NHDM provides an opportunity by improving data linkages between the NHM and PMJAY which will be a crucial step for extending access to primary care among Indian citizens and going toward UHC. This is in line with the HTA process where the HTA principles may be applied for interlinking databases and making them available at all levels for use in research and policy matters. Over the last few years, a few international fellowship programs on HTA (30) for the training of Health Care and Public Health Specialists have been conducted, supported by WHO India. The First HTA compendium was launched jointly by WHO Country Office and NHSRC (31), highlighting the most essential health technologies required to respond to the emerging disease burden in India.

The setting up of HTAIn happened around the same time the National Health Policy of 2017 was released, and the Ayushman Bharat Program 2018 was launched. The UHC is the common theme of these initiatives which has brought attention to the growing recognition of the need for priority setting in healthcare in India. With the vision to make UHC a reality, the Government of India has started several initiatives, development of Standard Treatment Workflows (STW) (32). HTA in the context of UHC and a step toward SDGs was a dedicated session in the 2019 World Conference on Access to Medical Products, held in November 2019 in New Delhi, India (33). With the setting up of HTAIn, the institutional mechanisms and capacity for HTA are increasingly being improved. Several resource centres across the country have been approved, and a few have already become functional. The academic institutions have been used as technical partners and capacity is being strengthened. The Technical Appraisal Committee (TAC) and Project Appraisal Committee (PAC) meet regularly, and more studies are being approved. To ensure the standardization of methods for undertaking HTA studies, a process manual has been prepared. Several training programs and workshops have been conducted to build the capacity of the RRCs and TPs.

Health Technology Assessment Board Bill, 2019 (34) has been put in place to institutionalize the structure and function of the HTAIn body. It would not only make innovative health tools reach patients faster, but also boost innovation and improve the competitiveness of the healthcare sector. Establishing a functioning system will create a policy demand for HTA outputs. HTA outputs may be linked with the explicit decision-making needs of UHC policies and budget impact analysis and allocation.

## 4. Discussion

The Ayushman Bharat Program of India, a vehicle to accelerate the progress of the country toward UHC, has two arms health and wellness centres (aiming to strengthen primary healthcare services) and Pradhan Mantri Jan Arogya Yojana (PMJAY), an insurance mechanism for secondary and tertiary hospitalization for bottom 40% population. HTA is being used extensively for both arms of ABP.

The Cost of Health Services in India (CHSI) study, a national-level study to support Ayushman Bharat-PMJAY Health Benefits Package Revision was carried out in 14 states. Finally, the AB-PMJAY package rates were revised in 2019, using the evidence on cost from the CHSI study. These HTA observations not only helped reduce the disparity between the cost and price for a given health benefits package, but also served to set the incentives for intended provider behavior in terms of provisioning of health care services. Similarly, a national EQ-5D Quality of Life valuation study was undertaken in six Indian States.

The use of HTA in primary health care services is paramount. Most topics evaluated as part of the HTAIn initiative have focused on choosing appropriate primary health care delivery strategies. This is even more relevant in a developing country such as India, where several known cost-effective interventions continue to have poor population coverage. As a result, more than the question of ‘whether’, it is ‘how’ should the service or intervention be delivered. In the same spirit, the HTAIn commissioned studies to find the most cost-effective way to screen for cervical cancer, diabetes and hypertension. Similarly, a few studies are being carried out to evaluate the appropriate family planning method, or method for the measurement of anaemia. Interestingly, the studies on screening resounded with an important message for primary health care – high coverage for the provision of subsequent treatment is an essential prerequisite for the cost-effectiveness of screening programs. This has significant implications for building strong comprehensive primary health care systems through creating functional health and wellness centres and strengthening the implementation of digital health technology.

Beyond ABP, health policy questions in wide areas of services are being addressed. An HTA report for evaluating the safety-engineered syringe for therapeutic use was used by the states of Punjab (35) and Andhra Pradesh for the introduction of SES in place of routine disposable syringes, well as aiding in the pricing decision. Secondly, in the context of the publicly financed cashless scheme for the treatment of hepatitis C in Punjab, the question of the use of a pan-genotypic drug velpatasvir for treatment was considered a priority. Accordingly, the study undertaken by the HTA which recommended the use of velpatasvir for cirrhotic HCV patients changed the standard treatment guideline, not only under the Punjab treatment program, but also the guidelines under the National HCV control program for the treatment of the disease (36).

Alongside, while there is a definitive role and need for health technology assessment, however, it is not a ‘solve all’ approach. Many of the health interventions are already proven and recommended for all settings. Nearly 80% of the health needs of the population can be addressed by available evidence, and no additional HTA is needed. Therefore, HTA studies should be carefully considered. Thailand and HiTAP is widely known for their work on HTA, and even in their settings, it is usually around a dozen of HTA studies which are done on annual basis. A challenge which many LMICs face in HTA is that while multidisciplinary skills are needed to assemble and interpret the data; however, countries with the greatest need often have the least capacity. Similarly, the lack of data available to arrive at decisions is often missing from these settings. Therefore, any attempt to strengthen HTA should have sufficient attention to the capacity building of many stakeholders. The mechanisms for strengthening the data collection and reporting and sufficient funding for improving data collection mechanisms and primary data collection need to be supported.

This article describes the UHC and HTA journeys and compiles a list of things to do in India. The findings will not be generalizable to other countries, but they will be useful in other similar contexts where they intend to use the HTA process for UHC.

## 5. Conclusion

Universal Health Coverage raises the need to choose and manage effective technologies and interventions to be adopted within countries’ health systems, particularly in a context of limited resources. Over the last few years, the mechanisms for HTA have significantly evolved in India, and additional institutional capacities for HTA are being developed. Several new HTA studies are being completed, and many more are in the process. Developing and strengthening national capacity will have to build on established best practices, information exchange and collaborative approaches to make the best use of limited resources and yield robust scientific assessments. Stronger mechanisms and capacity for HTA in India would contribute to accelerating the journey toward UHC in the country.

## Data availability statement

The original contributions presented in the study are included in the article/Supplementary material, further inquiries can be directed to the corresponding author.

## Author contributions

CL, TS, KR, and ShP conceptualized the study. CL and KS prepared the manuscript. All authors contributed to the article and approved the submitted version.

## Funding

This work was supported by the Health Technology Assessment in India (HTAIn), Department of Health Research, Ministry of Health and Family Welfare, Govt. of India.

## Conflict of interest

The authors declare that the research was conducted in the absence of any commercial or financial relationships that could be construed as a potential conflict of interest.

## Publisher’s note

All claims expressed in this article are solely those of the authors and do not necessarily represent those of their affiliated organizations, or those of the publisher, the editors and the reviewers. Any product that may be evaluated in this article, or claim that may be made by its manufacturer, is not guaranteed or endorsed by the publisher.
